# Diverse activation of microglia by chemokine (C-C motif) ligand 2 overexpression in brain

**DOI:** 10.1186/1742-2094-10-86

**Published:** 2013-07-17

**Authors:** Maj-Linda B Selenica, Jennifer A Alvarez, Kevin R Nash, Daniel C Lee, Chuanhai Cao, Xiaoyang Lin, Patrick Reid, Peter R Mouton, Dave Morgan, Marcia N Gordon

**Affiliations:** 1 Department of Molecular Pharmacology & Physiology, Byrd Alzheimer Institute, University of South Florida, Tampa, FL, USA; 2 Department of Pharmaceutical Sciences, College of Pharmacy, University of South Florida, Tampa, FL, USA; 3 Department of Pathology & Cell Biology, Byrd Alzheimer Institute, University of South Florida, Tampa, FL, USA

**Keywords:** BMDC, CCL2, Infiltration, Inflammation, Macrophage, Microglial activation, rAAV9

## Abstract

**Background:**

The chemokine (C-C motif) ligand 2 (CCL2) is a monocyte chemoattractant protein that mediates macrophage recruitment and migration during peripheral and central nervous system (CNS) inflammation.

**Methods:**

To determine the impact of CCL2 in inflammation *in vivo* and to elucidate the CCL2-induced polarization of activated brain microglia, we delivered CCL2 into the brains of wild-type mice via recombinant adeno-associated virus serotype 9 (rAAV-9) driven by the chicken β-actin promoter. We measured microglial activation using histological and chemical measurement and recruitment of monocytes using histology and flow cytometry.

**Results:**

The overexpression of CCL2 in the CNS induced significant activation of brain resident microglia. CD45 and major histocompatibility complex class II immunoreactivity significantly increased at the sites of CCL2 administration. Histological characterization of the microglial phenotype revealed the elevation of “classically activated” microglial markers, such as calgranulin B and IL-1β, as well as markers associated with “alternative activation” of microglia, including YM1 and arginase 1. The protein expression profile in the hippocampus demonstrated markedly increased levels of IL-6, GM-CSF and eotaxin (CCL-11) in response to CCL2, but no changes in the levels of other cytokines, including TNF-α and IFN-γ. Moreover, real-time PCR analysis confirmed increases in mRNA levels of gene transcripts associated with neuroinflammation following CCL2 overexpression. Finally, we investigated the chemotactic properties of CCL2 *in vivo* by performing adoptive transfer of bone marrow–derived cells (BMDCs) isolated from donor mice that ubiquitously expressed green fluorescent protein. Flow cytometry and histological analyses indicated that BMDCs extravasated into brain parenchyma and colabeled with microglial markers.

**Conclusion:**

Taken together, our results suggest that CCL2 strongly activates resident microglia in the brain. Both pro- and anti-inflammatory activation of microglia were prominent, with no bias toward the M1 or M2 phenotype in the activated cells. As expected, CCL2 overexpression actively recruited circulating monocytes into the CNS. Thus, CCL2 expression in mouse brain induces microglial activation and represents an efficient method for recruitment of peripheral macrophages.

## Background

Cerebral inflammation involving activation of resident glial cells, release of soluble mediators, such as cytokines and chemokines, and infiltration of peripheral bone marrow–derived myeloid cells (BMDCs) is a process that occurs throughout the course of many neurodegenerative diseases. The chemokine (C-C motif) ligand 2 (CCL2) protein (also known as macrophage chemoattractant protein 1, or MCP-1) is well-recognized for its ability to mediate macrophage recruitment and migration to sites of inflammation in rheumatoid arthritis, multiple sclerosis, traumatic brain injury, stroke and Alzheimer disease (AD) [1-7]. Transgenic mice that overexpress CCL2 in the central nervous system (CNS) exhibit a robust accumulation of macrophages and activation of microglia in the brain [8,9], whereas mice deficient in the *CCL2* gene show reduced leukocyte infiltration after autoimmune encephalomyelitis, traumatic brain injury, spinal cord injury and HIV-associated dementia [10-12]. In neurodegenerative diseases such as AD, recent evidence derived from genetically modified animal models suggests that infiltrating macrophages contribute to reduced amyloid pathology [13-18]. Other studies have indicated that CCL2 expression accelerates amyloidosis in an amyloid precursor protein (APP)/CCL2 bigenic mouse model, suggesting a different role of CCL2 in resident microglia and ultimately the AD pathology of these mice [19]. Also, CCL2 levels are reportedly increased in the brain, serum and cerebrospinal fluid of AD patients [20-22], further supporting a central role for this chemokine in neuroinflammation.

The impact of CCL2 on microglial activation and infiltration of specific macrophage subsets into the CNS may be very different in adult vs developing mice. Hence, in this experiment, we used recombinant adeno-associated virus (rAAV) to overexpress and distribute CCL2 in the CNS of adult mice and to investigate the effect of CCL2 on microglial activation *in vivo*. Microglia, the primary immune-competent cells of the CNS, exhibit many stages of activation and varying morphology [23,24]. Resting microglia exhibit a ramified morphology and express low levels of many cell surface immune molecules. Activated microglia exhibit an amoeboid morphology [25], increased phagocytic ability and enhanced migratory capacity within the brain, along with increased expression of cell surface glycoproteins, including CD45 and major histocompatibility complex class II (MHCII) [26]. The activation state of microglia is characterized by two main classes [27]. The “classically activated” M1 microglial phenotype is characterized by strong antigen-presenting abilities, proinflammatory cytokine production and the production of toxic intermediates such as nitric oxide and reactive oxygen species. The “alternatively activated” M2 microglia are involved in tissue repair and wound-healing processes, are characterized by high levels of arginase expression and are generated in response to cytokines such as IL-4, IL-10 and IL-13 [28,29].

Evidence for an essential role of CCL2 in macrophage activation in disease models such as atherosclerosis and asthma [5,30,31], together with the observation that inflammatory stimuli induce its expression, has led to consideration of CCL2 as an inflammatory chemokine. However, adaptive immunity studies have suggested that CCL2 expression is strongly associated with development of Th2 responses (for example, resistance in *Leishmania* major infection) [32] and with enhanced IL-4 and IL-10 secretion by T cells [33,34].

In this experiment, we (1) investigated the role of rAAV-delivered CCL2 on activation and polarization of microglia in the CNS microenvironment, (2) performed histological characterization of various activation states of microglia expressing M1 vs M2 activation markers and (3) measured extravasation of bone marrow–derived monocytes into the CNS in a CCL2-dependent fashion, without using the radiation required for bone marrow grafts. Interestingly, introduction of CCL2 via rAAV9 transduction resulted in secretion of cytokines representing both spectra of microglial activation states. Furthermore, real-time quantitative PCR demonstrated a dual effect of CCL2 on gene expression associated with microglial activation *in vivo*. We showed that a single chemokine can affect two very essential pathways, suggesting that CCL2 is an attractive target in neuroinflammatory diseases.

## Methods

### Experimental design

Wild-type, nontransgenic 14-month-old mice underwent intracranial injection of rAAV expressing CCL2 or green fluorescent protein (GFP), followed by adoptive transfer of GFP+ BMDCs. Mice were randomly assigned to two cohorts.

In cohort 1, mice were divided into two groups. The first group received unilateral, intracranial injections of rAAV9 vector expressing CCL2 (*n* =6) in both the right anterior cortex and right hippocampus. The second group received identically placed injections of rAAV9-GFP (control, *n* = 6). Seven weeks after the intracranial injections, mice received a single intracardiac injection of GFP+/CD11b+ bone marrow–derived monocytes (see below). The mice were killed 24 h later. Tissue from these animals was utilized for all immunohistochemical analyses presented in this report.

In cohort 2, mice of the first group received bilateral, intracranial injections of rAAV9-CCL2 (*n* = 6) in both the hippocampus and the anterior cortex for a total of four injections. The second group received identically placed injections of rAAV-GFP (*n* = 6). As described above, both groups received adoptive transfer of bone marrow cells 7 wk later. Half of the brain was collected and utilized for flow cytometry, and tissue from the other half of the brain was utilized for RT-PCR or multiplex assays as described below.

Studies conducted at our laboratory and others have demonstrated the capabilities of rAAV serotype 9 to transduce neurons within specific regions in the mouse brain [35,36]. rAAV9 has high transduction efficiency and drives robust gene expression. Gene expression occurs as early as 1 wk *in vitro* and *in vivo*[37,38] and persists for more than 9 mo in mouse brain [35]. Therefore, rAAV9 is a preferred candidate to deliver CCL2/GFP *in vivo*. We chose to examine microglial activation and monocyte recruitment 7 wk after viral injection to allow strong expression and to provide time for recovery from the intracranial injection.

A transgenic line ubiquitously expressing GFP was used as the source of bone marrow–derived monocytes (C57BL/6-Tg (UBC-GFP) 30Scha/J; The Jackson Laboratory, Bar Harbor, ME, USA). These transgenic mice express GFP under the direction of the human ubiquitin C promoter. All mice maintained in the University of South Florida Health Byrd Alzheimer Institute animal facility follow the according to guidelines established by “Guide for the care and use of laboratory animals” and are approved by IACUC.

### CCL2-AAV9 preparation

The CCL2 clone was generated from mouse cDNA and amplified using PCR with primers 5′- GAGACCGGTCCACCATGCAGGTCCCTGTCATGCTTC-3′ and 5′-GAGGCTAGCCTAGTTCACTGTCACACTGGTCACTCC-3′. CCL2 was cloned into the *Age*I and *Nhe*I sites of the rAAV vector pTR2-RMCS under the control of the hybrid cytomegalovirus chicken β-actin promoter. This vector also expresses red fluorescent protein (RFP) under the control of the thymidine kinase promoter and AAV2 terminal repeats. rAAV serotype 9 virus was generated using pAAV9 and pXX6 in HEK293 cells as described previously [39].

### Intracranial injections

The injection procedures were performed using a convection-enhanced delivery method described previously [39]. Briefly, mice were anesthetized with 1.5% isoflurane in 100% oxygen and secured into a stereotactic apparatus. Each animal was given an injection unilaterally (cohort 1, *n* = 6) or bilaterally (cohort 2, *n* = 6) with 2 μl of rAAV9-CCL2 (7 × 10^12^ vector genome (vg/ml) or 2 μl of rAAV9-GFP (control, *n* = 6 per cohort) into both cortex and hippocampus. The coordinates of injection were as follows: hippocampus (from bregma) anteroposterior –2.7 mm, lateral ±2.7 mm, dorsoventral −3.0 mm; cortex, anteroposterior (from bregma) +2.2 mm, lateral ±1.7 mm, dorsoventral −3.0 mm. A microsyringe injector and controller (Stoelting, Wood Dale, IL, USA) were used to inject 2 μl of virus at a constant rate of 2.5 μl/min in each placement. The needle was kept in place for 1 min after injection and then raised slowly. Mice were allowed to recover for 7 wk.

### Adoptive transfer of bone marrow–derived cells

CD11b+ cells were collected as previously described [40]. Briefly, transgenic mice ubiquitously expressing GFP were injected with a euthanasia solution, and their femurs and tibias were removed aseptically. Femoral and tibial marrow cavities were flushed with RPMI 1640 media containing fetal bovine serum and HEPES (4-(2-hydroxyethyl)-1-piperazineethanesulfonic acid), pH 7.4, using a 25-gauge needle. Single-cell suspensions were prepared by repeat pipetting, and the cell preparations were passed through a 70-μm nylon mesh to remove cell debris. Red blood cells (RBCs) were removed by exposure to 3 ml of RBC lysis buffer (5 min, room temperature) before dilution in cold phosphate-buffered saline (PBS). Cells were centrifuged, washed twice in RPMI 1640 and counted using a hemocytometer. CD11b+ cells were enriched using Miltenyi Biotec’s LS columns and MidiMACS magnet (Auburn, CA, USA) following the manufacturer’s instructions. Briefly, 70 million to 100 million bone marrow cells from GFP transgenic mice were suspended in 2.7 ml of PBS and 0.5% bovine serum albumin and incubated for 15 min, together with CD11b antibody conjugated to magnetic microbeads at 4°C. The cell suspension was applied to the supplied column in a magnetic field, and the CD11b+ fraction was separated from the unlabeled cells by washing three times with 3 ml of buffer. The column was separated from the magnet, and CD11b+ cells were collected. The purity of immunomagnetically separated cells was measured using a FACSCalibur flow cytometer (BD Biosciences, San Jose, CA, USA). Cells were then counted, and 5 × 10^6^ freshly isolated CD11b+ (GFP+) cells were resuspended in 100 μl of saline and injected into the left heart ventricles of CCL2- and rAAV9-GFP transduced mice of both cohort 1 and cohort 2.

### Tissue collection and histochemical procedures

Twenty-four hours after adoptive transfer, mice were killed and perfused transcardially with PBS. The brains from cohort 1 were removed and immersed in freshly prepared 4% paraformaldehyde in 100 mM phosphate buffer, pH 7.4, prior to being cryoprotected in a series of sucrose solutions, frozen and sectioned in the horizontal plane at 25 μm using a sliding microtome. Every eighth section was collected with a 50-μm thickness for stereology. Sections were stored at 4°C in Dulbecco’s PBS and 0.05% azide for immunohistochemistry. Immunohistochemistry was performed on free-floating sections as previously described [41]. A series of six sections from each animal were incubated with each primary antibody overnight at room temperature, then incubated in the biotinylated secondary antibody (2 h) followed by incubation for 1 h in avidin-biotin complex (VECTASTAIN Elite ABC kit; Vector Laboratories, Burlingame, CA, USA). Color development was performed using 1.4 mM diaminobenzidine with 0.03% hydrogen peroxide in PBS for 5 min. The following primary antibodies were used for immunohistochemistry: CCL2 (rat anti-CCL2; R&D Systems, Minneapolis, MN, USA), YM1 (rabbit anti-YM1; Stem Cell Technologies, Vancouver, BC, Canada), arginase 1 (chicken anti-Arg-1; generous gift from Dr Sidney Morris), IL-1β (rat anti-IL-1β; R&D Systems), S100A9 (goat anti-S100A9, R&D Systems), GFP (chicken anti-GFP; Abcam, Cambridge, MA, USA), CD45 (rat anti-mouse; AbD Serotec, Raleigh, NC, USA), MHCII (rat anti-MHCII; BD Biosciences). Immunofluorescence labeling was performed as follows. After incubation with the primary antibody, the free-floating sections were incubated for 2 h with the appropriate fluorophore-coupled secondary antibodies: Alexa Fluor 594 (1:1,500), Alexa Fluor 488 (1:1,500) or Alexa Fluor 405 (1:1,500; Invitrogen, Grand Island, NY, USA). Possible lipofuscin artefacts were quenched by treating slides with 3% Sudan Black B stain as described previously [42]. Sections were rinsed in Dulbecco’s PBS and coverslipped with VECTASHIELD HardSet Mounting Medium with or without 4′,6-diamidino-2-phenylindole (Vector Laboratories).

For cohort 2, the left hemisphere was dissected into brain regions and snap-frozen for biochemical analyses including RT-PCR and multiplex assay (see below). The right-half brain was immersed in Hanks’ balanced salt solution containing 0.2% collagenase type 3 (Roche Diagnostics, Mannheim, Germany) and 3.0 U/ml purified dispase (Worthington Biochemical Corp, Lakewood, NJ, USA) for 45 min at 37°C followed by incubation with 40 μg/ml DNase (Sigma, St Louis, MO, USA) for an additional of 15 min at 37°C. After a series of triturations, the cell suspension was mixed with physiological 90% Percoll solution and centrifuged as described previously [43]. Brain cells in suspension were then incubated with fluorescein isothiocyanate (FITC)-labeled antibody to GFP (1 mg/ml) (Abcam) or with isotype-matched control antibody for 30 min on ice. GFP fluorescence intensity was measured using a FACSCalibur flow cytometer and analyzed using BD CellQuest Pro software (BD Biosciences).

### Image analysis and stereology

Immunohistochemistry was quantified using Image-Pro Plus software (Media Cybernetics, Rockville, MD, USA). Segmentation of positive stain was performed using RGB identification and was held constant throughout the immunohistochemical analysis. The hippocampus was manually circumscribed and the percentage area of immunostaining was calculated in each hemisphere. A similarly sized area at the site of the rAAV placement in the anterior cortex was analyzed separately. Values obtained from all sections from a single mouse were averaged to represent a single value for that animal (per brain region and per hemisphere).

Stereological analysis was performed as previously described [44]. We used a rare event protocol in which all positive cells in the region analyzed were counted as follows after confirming they were not visible on the upper surface of the section: *N* = (number of cells counted) × (1/*ssf*) × (1/*asf*) × (1/*tsf*), where *ssf* is the section sampling fraction (that is, one-eighth of the total sections used), *asf* is the area sampling fraction (in this case, the entire region rather than a dissector, thus *asf* = 1) and *tsf* is the thickness sampling fraction (*tsf* = 1).

### Multiplex chemokine/cytokine assay

Snap-frozen left hippocampi from the animals in cohort 2 were homogenized in radioimmunoprecipitation assay buffer (50 mM Tris, pH 7.5, 150 mM NaCl, 1 mM ethylenediaminetetraacetic acid, 1% Triton X-100, protease inhibitor cocktail and phosphatase inhibitor cocktail I and II; Sigma). The protein concentration of each sample was measured using the Bradford protein assay (Bio-Rad Laboratories, Hercules, CA, USA) and adjusted to 4.5 mg/ml. The concentrations of IL-1β, IL-4, IL-6, IL-10, IL-12p70, IL-13, IL-17, interferon γ (IFN-γ), tumor necrosis factor α (TNF-α), CCL2, eotaxin 1 (CCL11) and granulocyte macrophage colony-stimulating factor (GM-CSF) were measured using the mouse cytokine/chemokine panel (MILLIPLEX MAP kit; Millipore, Billerica, MA, USA) according to the manufacturer’s protocol. In brief, the Bio-Plex Suspension Array System (Bio-Rad Laboratories) was calibrated using CAL2 with the high PMT setting of the Bio-Plex calibration kit, and standard sample preparation was performed according to the manufacturer’s directions. The filter plate was prewetted with wash buffer and vacuum-filtered before adding standard, control or study samples to the appropriate wells. Mixed capture beads were then added to each well, and plates were incubated overnight at 4°C with shaking. After two washes, 25 μl of detection antibody were added to each well, incubated for 1 h at room temperature and then treated with 25 μl of streptavidin-phycoerythrin for 30 min at room temperature. The plate was washed twice, and 150 μl of the Bio-Plex sheath fluid assay buffer were added to each well and read using the Bio-Plex Suspension Array System software (Bio-Rad Laboratories) per the kit instructions. The concentration of each analyte was calculated according to the standard curve.

### Quantitative real-time PCR

Total RNA from mouse anterior cortex (cohort 2) was isolated using the RNeasy kit (QIAGEN, Valencia, CA, USA) and reverse-transcribed using SuperScript III reverse transcriptase (Invitrogen, Carlsbad, CA, USA). Gene transcript functions included in this study (Table 1) have been reported previously [45-58] and were recently published by investigators at our laboratory [59].

**Table 1:** Expression of gene transcripts in mice overexpressing CCL2 compared with control mice

**Accession_ID**	**Gene symbol**	**Description**	**Fold change CCL2/Control**	***P*-value**	**Function**
**M1 genes**					
NM_010766	*Marco*	Macrophage receptor with collagenous structure	0.6 ↓	*P* < 0.004	Scavenger receptor expressed in a subset of macrophages [39]
NM_001002898	*Sirpβ*	Signal-regulatory protein β	17.13↑	*P* < 0.002	Cytoskeleton rearrangement, counterregulates proinflammatory mediators, phagocytosis [40]
NM_013650	*S100A8*	S100 calcium-binding protein A8 (calgranulin A)	3.1↑	*P* < 0.03	Calcium-binding protein involved in innate immune response [41]
NM_009114	*S100A9*	S100 calcium-binding protein A9 (calgranulin B)	ND		Forms heterodimeric protein complex with S100A8 [43,44]
NM_011593	*Timp1*	Tissue inhibitor of metalloproteinase 1	ND		Inhibitory role against most matrix metalloproteinases, promotes cell proliferation [45,46]
NM_010927	*iNOS*	Nitric oxide synthase, inducible	ND		Immune defense against pathogens, induces inflammation and oxidative stress [47]
NM_001111274	*IGF-1*	Insulin-like growth factor 1	ND		Mammalian growth and development [48]
**M2 genes**					
NM_007482	*Arg1*	Arginase 1, liver	2.4↑	*P* < 0.006	Arginine and proline metabolism [49]
NM_007695	*Chi3l3*	Chitanase 3-like 3	2.7↑	*P* < 0.003	Expressed in myeloid precursor cells, involved in inflammation (alternative activated macrophages) [27]
NM_020509	*Retnla*	Resistin-like α	ND		Alternatively activated macrophages and tissue repair [50]
**Chemotactic (M2) genes**					
NM_019577	*CCL24*	C-C motif chemokine 24	1.7↑	*P* < 0.04	Chemotaxis [51]
NM_001013412	*CCL26*	C-C motif chemokine 26	ND		Induces migration of eosinophils macrophages and basophils [52]
NM_007894	*EAR1*	Eosinophil-associated, ribonuclease A family, member 1	ND		RNase activity, chemotactic for eosinophils, alveolar macrophages [53]
NM_007895	*EAR2*	Eosinophil-associated, ribonuclease A family, member 2	3.8↑	*P* < 0.02	RNase activity, chemotactic for eosinophils, alveolar macrophages [53]
NM_053113	*EAR11*	Eosinophil-associated, ribonuclease A family, member 11	ND		RNase activity, chemotactic for eosinophils, alveolar macrophages [53]

qRT-PCR was performed in anterior cortex brain homogenate from animals injected with rAAV9-GFP (*n* = 6) and rAAV9-CCL2 (*n* = 6). RNA (10 ng/μl) of each sample was used for cDNA synthesis. The mass values for the genes of interest were normalized to glyceraldehyde 3-phosphate dehydrogenase (GAPDH) mRNA to determine fold change in mRNA expression relative to the standard RNA pool. The fold-change values for samples in rAAV9-GFP-treated vs rAAV9-CCL2-treated animals were analyzed and presented as M1- and M2-related gene transcript expression. A cycle threshold change (ΔCt) greater than 36 cycles was considered nondetected (ND). Statistical analyses were performed using Student’s *t*-test (*n* = 6). *P* values were specified for each gene. ↑ and ↓ represent significant increases or decreases from control mice at *P* < 0.05. ND, not detected. (Please contact the authors for details regarding genes).

QuantiTect primers for each gene (*Arg-1*: cat. no. QT00134288, *Retnla*: cat. no. QT00254359, *Marco*: cat. no. QT00102004, *S100A8*: cat. no. QT01749958, *S100A9*: cat. no. QT00105252, *Timp1*: cat. no. QT00996282, *Sirpβ*: cat. no. QT02332008, *Chi3l3*: cat. no. QT00108829, *iNOS*: cat. no. QT00100275, *CCL24*: cat. no. QT00126021, *CCL26*: cat. no. QT01559481, *EAR1*: cat. no. QT00261303, *EAR2*: cat. no. QT00265965, *EAR11*: cat. no. QT00266959, *IGF-1*: cat. no. QT00154469) were purchased and used for SYBR Green-based real-time RT-PCR (QIAGEN). qPCR was performed as follows: one cycle at 95°C for 5 min, one cycle at 95°C for 30 s and one cycle at 60°C for 1 min followed by 40 amplification cycles at 95°C for 30 s. The melting curve readings were performed every 1°C at 56°C to 99°C, holding for 1 s between readings. The DNA Opticon 2 System for Real-Time PCR Detection Version 4.3 (Bio-RadLaboratories) was used to detect the amplification products. None of the primers demonstrated more than one peak of fluorescence in melt curve analysis. The standard curve was calculated by plotting the cycle threshold change (∆Ct) against the log nanogram quantity of RNA added [60]. Linear regression was performed, and the slope relating ∆Ct to log nanograms of RNA was calculated and converted to a mass quantity of standard RNA. The mass values for the genes of interest were then normalized to GAPDH mRNA to determine the fold change in mRNA expression relative to the standard RNA pool. A ∆Ct cut-off greater than 36 cycles was considered nondetectable (ND).

### Statistical analysis

Statistical analyses were performed using Student’s *t*-test or one-way analysis of variance (ANOVA) followed by Fisher’s least significant difference *post hoc* means comparison test using StatView version 5.0 software (SAS Institute Inc, Cary, NC, USA). Graphs were generated using GraphPad Prism 4.0 software (GraphPad Software, La Jolla, CA, USA).

## Results

### CCL2 expression was exacerbated in rAAV9-CCL2 injected mice

**Figure 1:**
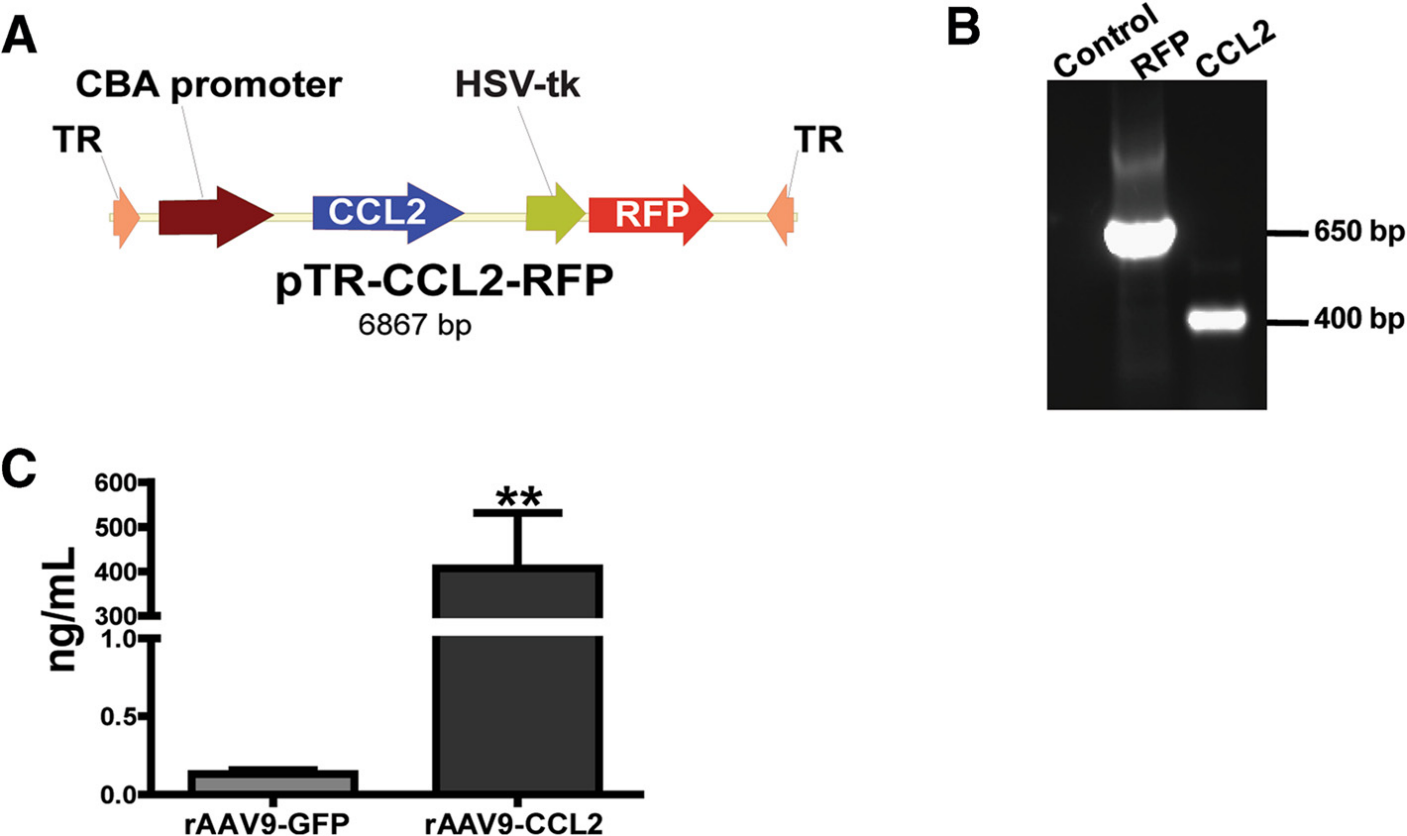
**Production of rAAV-CCL2 construct and documentation of CCL2 overexpression in mouse tissue. (A)** Schematic representation of pTR2-GMCS vector construct used to express chemokine (C-C motif) ligand 2 (CCL2). Chicken β-actin (CBA) promoter was used to drive the expression of CCL2, and RFP expression was driven by HSV thymidine kinase (HSV-tk) promoter. Construct vector was flanked by two terminal repeats (TR). **(B)** PCR product of the expected size for CCL2 (440 bp). **(C)** Expression of CCL2 (mean ± SEM, ng/ml) in the hippocampus 7 wk after injection of animals with either rAAV9-CCL2 or rAAV9-GFP (**P < 0.01, *n* = 6).

**Figure 2:**
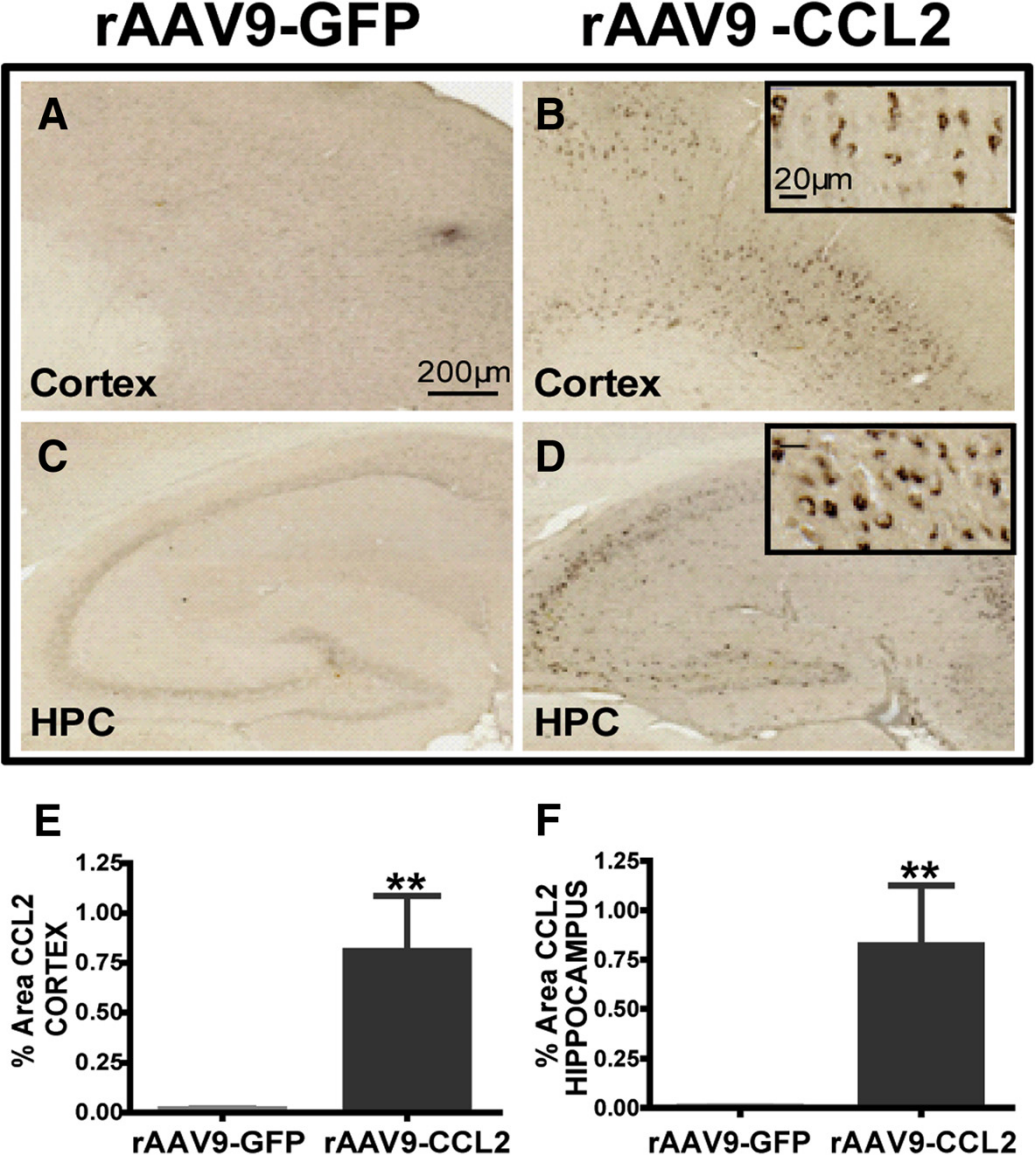
**CCL2 distribution and expression in the brain.** Immunohistochemical staining for CCL2 was performed. **(A)** through **(D)** Overall distribution of CCL2 in the brains of animals injected with rAAV9-GFP and rAAV9-CCL2. Photomicrographs represent the expression of CCL2 in the ipsilateral (injected) hemisphere in GFP control **(A)** and **(C)** and CCL2 animals **(B)** and **(D)**. Higher magnification shows neuronal localization of CCL2 expression (insets). **(E) **and **(F)** present the means ± SEM of percentage positive area of immunostaining for CCL2 in the cortex and hippocampus, respectively. Scale bar represents 200 μm (20 μm for insets). Statistical analyses were performed using Student's *t*-test (**P < 0.01, *n* = 6 per group).

To assess the role of CCL2 in activation of resident brain glial cells and infiltration of peripheral macrophages, we constructed a rAAV-9 pTR2-CCL2 vector (Figure 1A). The PCR product of RFP and mouse CCL2 is shown in Figure 1B (650 bp and 440 bp, respectively). Fourteen-month-old mice were injected with either rAAV9-CCL2 or rAAV9-GFP viral particles, unilaterally (cohort 1, see Methods) or bilaterally (cohort 2) in both the hippocampus and the cortex. CCL2 protein levels were measured in the hippocampal tissue of cohort 2 animals at 7 wk after the injection by using the MILLIPLEX MAP Multiplex assay kit (Figure 1C). Basal levels of CCL2 in rAAV9-GFP-injected animals were in the low range (0.13 ng/ml); however, as expected, CCL2 expression was significantly higher in the hippocampi of rAAV9-CCL2-injected animals (406 ng/ml). In addition, we confirmed expression of CCL2 by immunohistochemical staining (Figure 2). CCL2 expression was observed throughout the ipsilateral hippocampus and cortex of animals injected with rAAV9-CCL2 (Figures 2B and 2D). As expected, we did not detect CCL2 immunoreactivity in the mice injected with rAAV-GFP (Figures 2A and 2C) or in the contralateral hemisphere (not shown). The area occupied by CCL2 immunoreactivity was significantly larger in mice injected with rAAV-CCL2 than in mice injected with rAAV-GFP (Figures 2E and 2F).

### CCL2 is chemotactic after adoptive transfer of CD11b-positive bone marrow–derived cells

**Figure 3:**
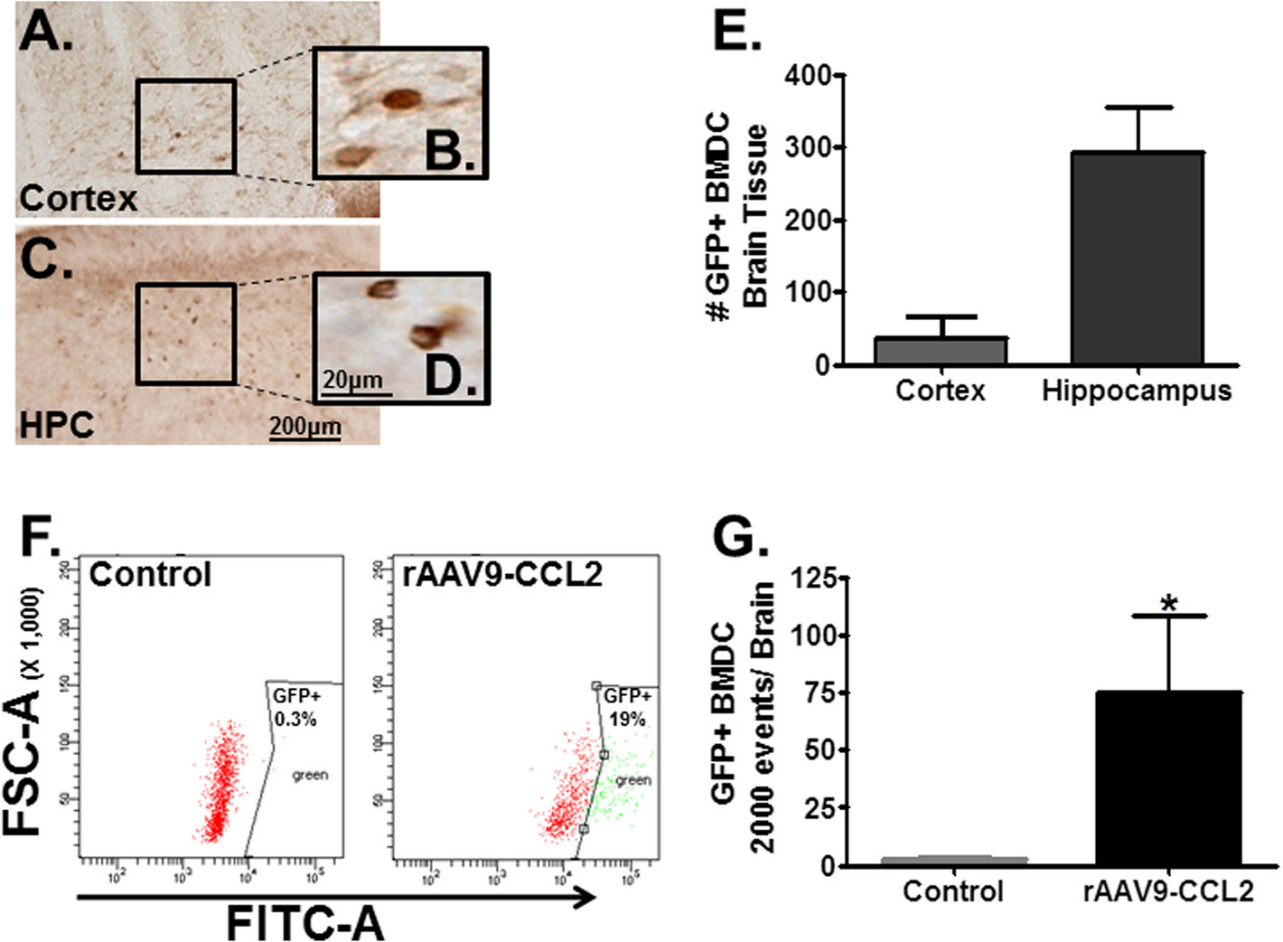
**CCL2 overexpression induces GFP+ BMDC extravasation into mouse CNS parenchyma.** Five million CD11b+ BMDCs were injected into the left ventricles of rAAV9-CCL2 mice. Photomicrographs of GFP+ infiltrating cells in the cortex **(A)** and **(B)** and hippocampus **(C)** and **(D)** are presented. Insets in **(B)** and **(D)** represent higher magnification of GFP+ cells. **(E)** The number of infiltrating GFP+ BMDCs was determined by cell counting in both hippocampus and cortex. CCL2 overexpression in hippocampus resulted in significantly more GFP+ cells than in the cortex in this cohort. **(F)** Representative data set from flow cytometry profile of one of six mice per group analyzed to detect the GFP+ cells infiltrating the brain homogenate. The percentages of cells were derived from data gated on lower forward-scatter light characteristics versus FITC fluorescence intensity. **(G)** Data analysis of 2,000 cells from flow cytometry where cells with fluorescence intensity greater than 1 × 10^4^ were counted and graphed using GraphPad Prism 5 software (GraphPad Software). Student’s *t*-test, *P < 0.05, **P < 0.01. Scale bars represents 200 μm and 20 μm.

To investigate the role of CCL2 in the chemotaxis of peripheral myeloid cells in the brain, we systemically injected CD11b+ BMDCs isolated from donor mice and monitored their uptake into the CNS. The BMDCs ubiquitously overexpressed GFP. Twenty-four hours before tissue collection, 5 million CD11b+/GFP cells (mostly monocytes) in a volume of 100 μl were injected into recipient mice previously treated with rAAV9-CCL2 or rAAV9-GFP (cohort 1 or 2). Using both histological evaluation and flow cytometry, we confirmed the migration of these cells into the CNS of CCL2-injected mice (Figure 3). To identify the extravasation and distribution of GFP+ cells, we first performed immunohistochemical staining for GFP using sections from rAAV9-CCL2-injected animals (cohort 1). Our data showed that GFP+ BMDCs were located mainly in the parenchyma of the ipsilateral hippocampus and cortex as well as in the perivascular area (Figures 3A through 3D). Furthermore, we used unbiased stereology [44] to count CD11b+/GFP+ labeled cells in the CNS after overexpression of CCL2 (Figure 3E). More GFP+ cells were counted in the ipsilateral hippocampus compared to the ipsilateral cortex. GFP+ cells in the rAAV-GFP-injected mice could not be counted, because there was marker overlap between the rAAV-GFP and the GFP+ monocytes.

In addition, we used flow cytometry (Figures 3F and 3G). The right hemispheres of animals in cohort 2 were processed to isolate CNS single-cell suspensions from the CCL2- and GFP-injected mouse brains as described previously [43]. This method resulted in collection of cells based on their forward-scatter parameter. The isolated cells were stained with FITC-conjugated anti-GFP antibody. No GFP+ cells were recovered from animals treated with rAAV-GFP, suggesting that transfected neurons are not recovered using this methodology. The GFP+ population (green) identified by the increased FITC intensity (boxed area) was significantly enriched as a proportion of the CNS cells in CCL2-injected mice compared with controls (19% vs 0.3%, respectively) (Figure 3F). Analysis of 2,000 events from each brain preparation revealed that GFP+ cells found in the CCL2-injected mice were significantly higher than in the control animals (Figure 3G).

### CCL2 induces CD45 microglial activation

**Figure 4:**
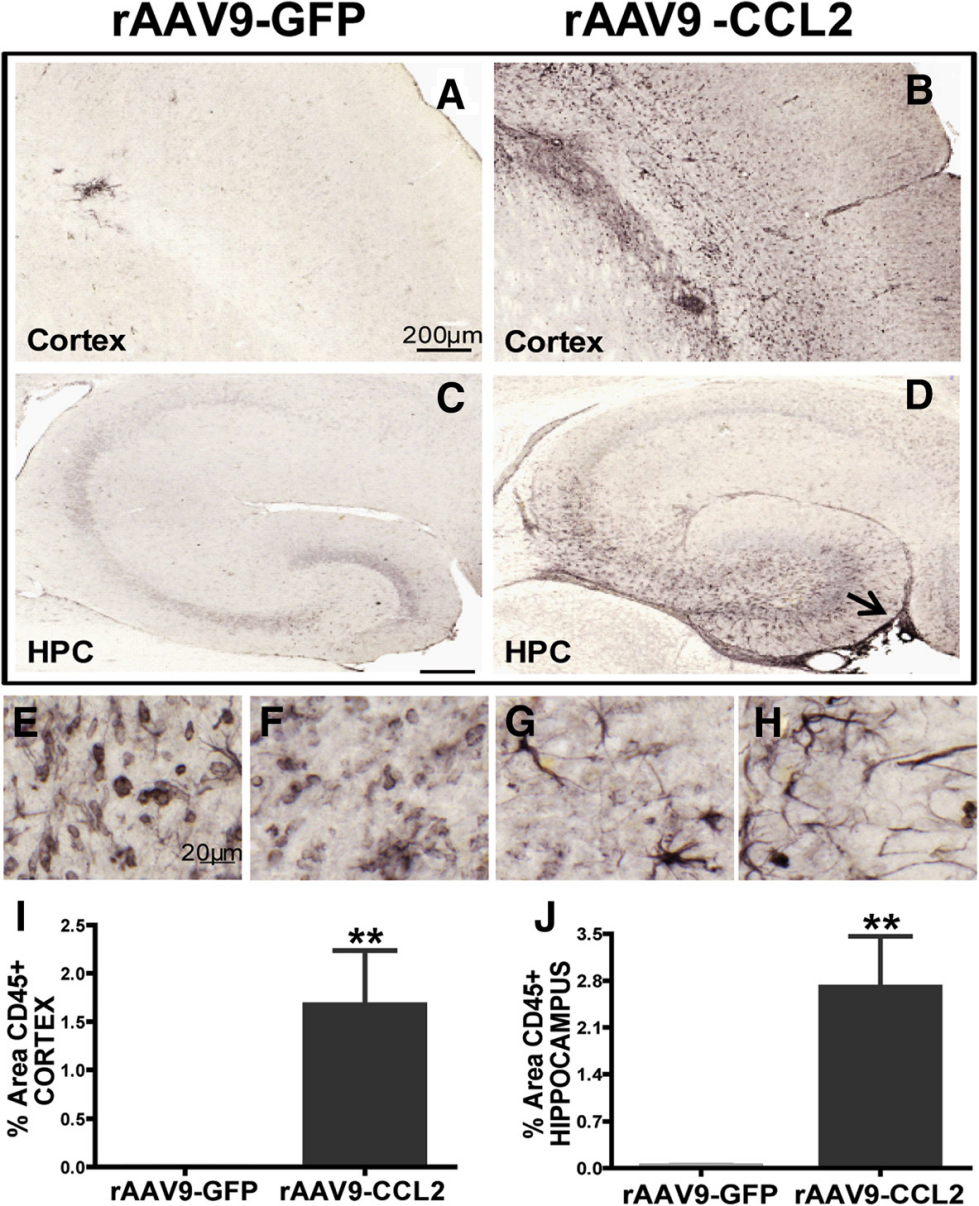
**CD45 accumulation in the brain is driven by CCL2 overexpression. (A)** through **(D)** Immunohistochemical staining for CD45 revealed significantly increased activation/recruitment of CD45+ macrophages in the cortex **(B)** and hippocampus **(D)** of rAAV9-CCL2-injected animals, but not in the control group **(A)** and **(C)**. CD45+ macrophages display various phenotypic morphologies in rAAV9-CCL2 animals **(E)** through **(H)**. Quantification of the percentage area containing positive CD45 immunostaining is presented for each brain region in **(I)** and **(J)** (mean ± SEM, *n* = 6). Statistical analysis was performed using Student's *t*-test (**P < 0.01). Scale bars represent 200 μm **(A)**, **(B)**, **(C)** and **(D)** and 20 μm **(E)**, **(F)**, **(G)** and **(H)**.

The contribution of CCL2 in regulating leukocyte accumulation in the CNS following CCL2 transduction was assessed by immunohistochemistry. To verify the effect of CCL2 expression on activation of microglia, immunoreactivity of brain tissue for CD45 + microglia was measured in rAAV9-CCL2 and rAAV9-GFP injected mice. Accumulation of mononuclear cells positive for CD45 in the brain 7 weeks post rAAV injection were observed in both hippocampus and cortex of CCL2 overexpressing animals (Figure 4). CD45 mononuclear cells occupied areas around the injection site and in the blood vessels lining ipsilateral hippocampus (arrow, Figure 4D, E and F). Following CCL2 overexpression, microglia were activated and acquired a ramified morphology throughout both cortex and hippocampus (Figure 4B, D, G and H). CD45+ microglia levels were significantly higher in the parenchyma of the ipsilateral hemisphere in CCL2 injected mice compared to the GFP control mice. When quantified using image analysis (percent positive area), we found that CD45 expression was significantly increased in cortex and in the hippocampus of CCL2 overexpressing mice (Figure 4I and J). Microglia from rAAV9-GFP injected mice remained negative for CD45 expression in both regions of interest (Figure 4A, C and I-J).

### Microglia exhibit enhanced expression of cell-surface MHCII following CCL2 overexpression

**Figure 5:**
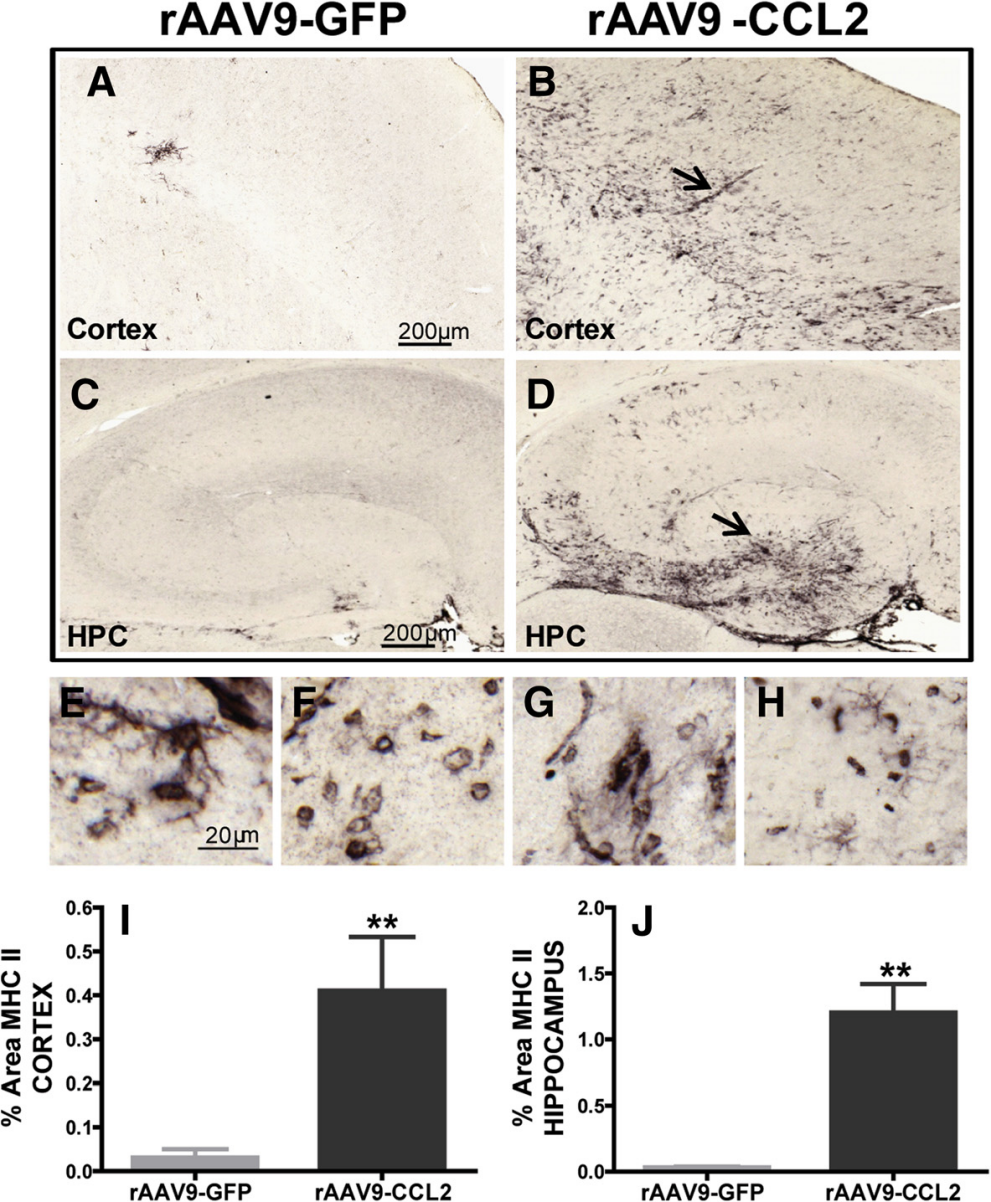
**MHCII activation is induced in the brains of CCL2-overexpressing mice. (A)** through **(D)** Immunohistochemical staining for MHCII showed that mononuclear cells/macrophages significantly increased MHCII expression following CCL2 overexpression. Images were collected from the cortices and hippocampi of wild-type mice injected with either rAAV9-GFP **(A)** and **(C)** or rAAV9-CCL2 **(B)** and **(D)**. MHCII+ cells displayed various phenotypic morphologies, from rounded to ramified (arrow) and amoeboid (arrowhead) **(E)** through **(H)**. Mean ± SEM percentage area for immunostaining of MHCII+ microglia in cortex and hippocampus are presented in **(I)** and **(J)**. Student's t-test. (**P < 0.01, *n* = 6). Scale bars represent 200 μm and 20 μm.

To further characterize the effect of CCL2 on brain microglial activation and phenotype surface molecules affected by this stimulus, we investigated the expression of MHCII (Figure 5). MHCII expression is detected on activated brain resident microglia, as well as on infiltrating immature myeloid cells, which can differentiate into MHCII+ macrophages [61]. CCL2 increased the accumulation of cells that were highly immunoreactive for MHCII in the ipsilateral cortex and hippocampi of rAAV-CCL2-injected animals (Figures 5B and 5D through 5H). These MHCII cells presented various morphologies, including the ramified morphology typical of activated microglia (higher magnification shown in Figures 5E and 5H, arrows), although other cells had a more rounded morphology consistent with perivascular macrophages (Figures 5F and 5G, arrowheads). We observed that MHCII+ round cells accumulated near the sites of injection, such as the dentate gyrus in the hippocampus, blood vessels in the hippocampus and vessels descending from the pia in the cortex (Figures 5B and 5D, arrows). We were unable to detect MHCII immunoreactivity in the brain microglia of animals transduced with rAAV9-GFP, except in the immediate vicinity of the injection site (Figures 5A and 5C). Quantification of MHCII expression by image analysis showed significantly higher immunoreactivity in both the cortex and the hippocampus of rAAV-CCL2-treated mice vs control mice (Figures 5I and 5J).

### CCL2 overexpression leads to induction of classical microglial activation (M1 state)

**Figure 6:**
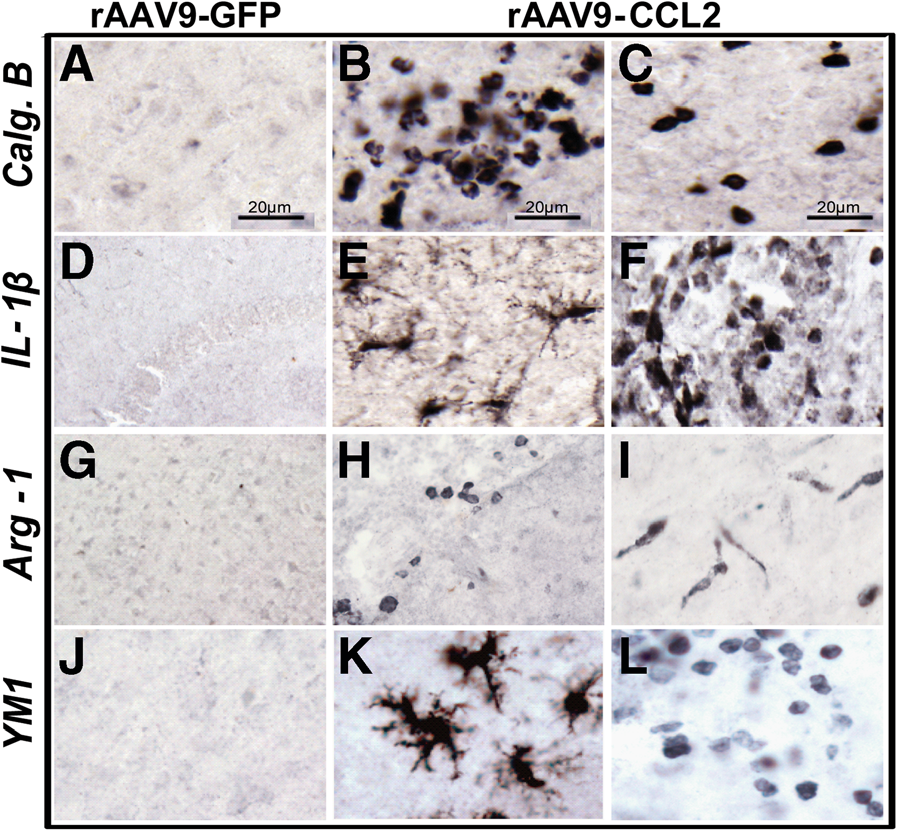
**Induction of microglial markers of classical activation (M1) and alternative activation (M2) following rAAV9-CCL2 transduction.** Mice which received control GFP viral vector **(A)**, **(D)**, **(G)** and **(J)** or mice overexpressing CCL2 **(B)**, **(C)**, **(E)**, **(F)**, **(H)**, **(I)**, **(K)** and **(L)** were stained using immunohistochemistry for microglial markers representing the M1 activation state: calgranulin B (Calg.B or S100-A9: **(A)**, **(B)** and **(C)**) and IL-1β (**(D)**, **(E)** and **(F)**); or the M2 activation state: arginase 1 (Arg-1: **(G)**, **(H)** and **(I)**) and YM1 **(J)**, **(K)** and **(L)**. **(C)**, **(F)**, **(I)** and **(L)** are images of different microglial morphology stained for each marker. Scale bar is 20 μm.

To this point, our data suggested that CCL2 overexpression can generate activation of microglia using markers such as CD45 and MHCII. CCL2 has been reported to be a pro-inflammatory chemokine, with its primary effect on the innate immune system via chemotaxis of monocytes. However, a few studies have reported that CCL2 affects the adaptive immune system by Th1/Th2 polarization [32,33]. Therefore, we investigated the effect of increased CCL2 levels on the CNS microglia activation stages, also referred to as M1/M2 activation. To understand the effect of CCL2 on polarization of brain microglia, we evaluated markers of microglial classical activation (Figures 6A through 6F). Both IL-1β and calgranulin B (S100A9) are proinflammatory proteins that are associated with classical activated microglia. S100-A9 is an inflammatory marker protein expressed almost exclusively in myeloid cells [62]. We found that CCL2 induced high levels of S100-A9 expression and that S100-A9 positive cells were prominently located around the site of injection. The positive cells were exclusively of the rounded mononuclear phenotype (Figure 6B and C). These findings suggest that the S100A9-expressing cells did not transform into an activated phenotype, in spite of the CCL2 stimulus, or that the observed mononuclear phenotype represents the newly recruited cells responding to the CCL2 stimulus [63]. Immunohistochemical evaluation revealed IL-1β positive microglia, found in the parenchyma of the ipsilateral hemisphere of CCL2 injected mice. The parenchymal IL-1β positive cells displayed predominantly a ramified/activated phenotype (Figure 6E) although rounded mononuclear cells were observed in the vicinity of blood vessel and meninges (Figure 6F). Brain tissue from control animals did not stain positively for any of the M1 markers (Figures 6A and 6D).

### CCL2 overexpression induces expression of alternative activation (M2) microglia markers and regulates cytokine expression

To determine whether CNS overexpression of CCL2 alters microglial activation toward an alternative activation (M2) state, we performed histological evaluations using arginase 1 (Arg-1) and chitinase 3-like-3 (YM1) as specific markers for the M2-activated microglia [64]. The pattern of inflammatory cells was different in rAAV9-CCL2-injected mice than in rAAV-GFP-injected control mice (Figure 6). The microglia of CCL2-overexpressing mice were immunoreactive for the Arg-1 marker in the ipsilateral hemisphere, and the Arg-1+ microglia showed a range of morphologies (Figures 6H and 6I). For instance, we observed Arg-1+ microglia displaying a rod-shaped phenotype (Figure 6I), whereas Arg-1+ rounded mononuclear cells were occasionally found in the CCL2 mice (Figure 6H). YM1 represents another alternative activated microglial cell–surface marker. We found that CCL2 overexpression induced YM1 expression on brain microglia, and, overall, YM1+ cells were present in the regions where overexpression of CCL2 was more abundant. YM1+ microglia displayed equal distribution between ramified and rounded morphologies in both brain regions (Figures 6K and 6L). We found that rAAV9-GFP animals did not show positive immunoreactivity for any of the alternative activation microglial markers (Figures 6G and 6J). These results suggest that microglia adopted the M2 phenotypic characteristics in response to CCL2 overexpression.

**Figure 7:**
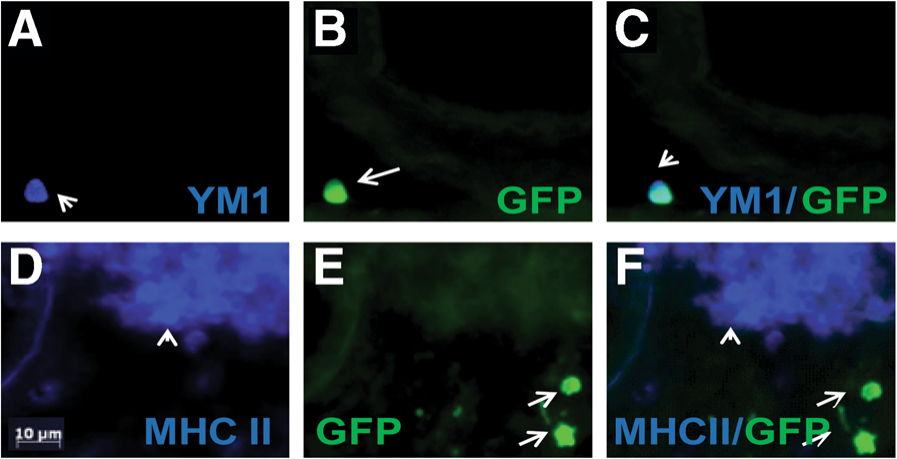
**Recruited BMDCs express some markers in common with microglia. (A)** through **(C)** show immunofluorescent staining of YM1 **(A)**, blue on a recruited GFP BMDC **(B)**, green in the blood vessels surrounding the hippocampus of a CCL2-overexpressing animal. **(D)** through **(F)** In contrast, recruited cells **(**GFP, **E)** did not express MHCII **(D**, blue**)**. Merged images of each respective double-stain are shown in **(C)** and **(F)**. Scale bar represents 10 μm.

Next, we performed double-immunofluorescence to determine whether infiltrating GFP+ BMDCs colocalized with markers of activated microglia. We found that some infiltrating GFP+ cells that extravasated into the parenchyma of the CCL2-treated mouse brain colabeled with YM1 (Figures 7A through 7C) and CD45 (data not shown). On the other hand, recruited GFP+ cells did not colabel with MHCII (Figures 7D through 7F). Consequently, markers currently in use to identify microglia fail to discriminate between intrinsic and newly recruited cells.

### AAV9-mediated CCL2 overexpression induces protein and mRNA changes in genes associated with neuroinflammation

**Figure 8:**
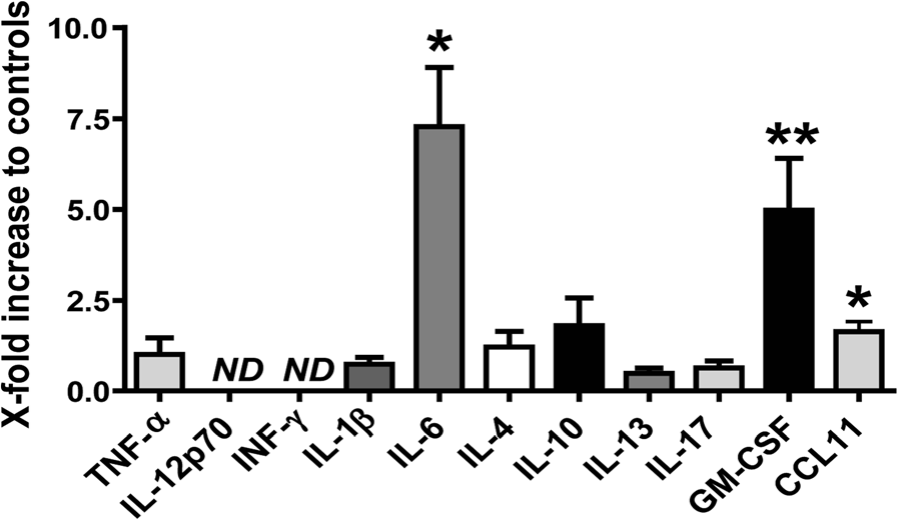
**Induced protein expression profile following CCL2 overexpression.** A series of cytokine and chemokine protein expression levels were measured using the mouse multiplex assay in hippocampal tissue after rAAV9-CCL2 transduction. Cytokine levels are expressed as fold changes relative to the GFP control animals (*n* = 6). *P < 0.05, **P < 0.01.

Next, we assessed the CCL2-induced production of cytokines reported to be indicative of M1 vs M2 activation states. Hippocampi from rAAV9-CCL2- and rAAV9-GFP-injected animals (cohort 2) were homogenized and processed as described above. We observed nine- and sixfold increases in the levels of IL-6 and GM-CSF (Figure 8), respectively, in the hippocampi of CCL2-treated mice (5.8 ± 1.9 pg/ml vs 7.5 ± 3.9 pg/ml) compared to GFP-injected mice (0.5 ± 0.2 pg/ml vs 1.2 ± 0.4 pg/ml). In addition, eotaxin 1 (CCL-11) levels were increased 2.5-fold in response to CCL2 overexpression (13.9 ± 3.4 pg/ml vs 7.5 ± 0.8 pg/ml, *P* < 0.05, in CCL2-injected animals vs GFP control animals, respectively). Meanwhile, CCL2 overexpression did not significantly increase the levels of the M1-related cytokines TNF-α, IL-1β and IL-17, whereas levels of IL-12p70 and IFN-γ were undetectable. In addition, mean levels of M2-related cytokines IL-4, IL-10 and IL-13 were slightly increased in the CCL2-injected mice but these data did not reach statistical significance (26 ± 5.1 pg/ml, 12 ± 3.6 pg/ml and 392 ± 207 pg/ml, respectively) compared to control animals (14 ± 5.6 pg/ml, 7.2 ± 3.0 pg/ml and 482 ± 396 pg/ml, respectively). In summary, CCL2 overexpression in mice produced a unique pattern of inflammatory signals, with significant inductions in GM-CSF, eotaxin 1 and IL-6, at the time of investigation.

We used quantitative RT-PCR to investigate CCL2-induced changes in the mRNA levels of gene transcripts that are associated with M1 or M2 activation states [27,59]. We used the anterior cortex region of animals from both groups of cohort 2 to identify the genes that were differentially expressed. We used the rigorous criteria of qRT-PCR described previously [65] to determine that 7 of 14 gene transcripts were significantly changed by overexpression of CCL2 (Table 1). We showed a significant increase in gene transcripts such as Arg-1, Chi3L3 (YM1) and EAR2 (2.4-, 2.7- and 3.8-fold increases, respectively). These genes and their respective proteins are widely used as markers of M2 alternatively activated macrophages and are generally responsive to the effects of cytokines such as IL-4 and IL-13 [64]. Gene transcripts associated with M1 classically activated microglia were also induced. S100A8 and S100A9 are two small calcium-binding proteins increased during proinflammatory activity. The expression of S100A8 was increased threefold in the cortex of CCL2-overexpressing animals compared to controls. The expression of the Sirp1β transcript, a member of transmembrane glycoproteins expressed in myeloid cells and associated with phagocytosis [66], was strongly (17-fold) increased in response to CCL2. Because CCL2 overexpression significantly increased levels of eotaxin 1 protein, we investigated the expression levels of other C-C motif ligands with chemotactic activity. We found a 1.7-fold increase in CCL24 (eotaxin 2), but not in CCL26 (eotaxin 3, Table 1), compared to control tissue. Not all the genes related to the M1 or M2 activation state of microglia were affected by CCL2 (Table 1). For example, expression of iNOS, which is involved in the M1 classical activation of microglia, was not changed. Marco expression, another M1 marker, declined slightly, whereas the expression of S100A9 RNA was unchanged, despite the increased protein observed by immunohistochemical analysis. These findings suggest that the effect of CCL2 can be specific to some of the gene transcripts, but not to all microglia-related gene transcripts.

## Discussion

Infiltration of peripheral macrophages into the CNS occurs in small quantities, and it is not clear what role, if any, they play in age-associated neurodegenerative disease. In addition, whether their effect is beneficial or pathological appears to be dependent on the CNS insult (for review, see [67]). Less attention has been focused on the responses of resident brain microglia to CCL2. Consequently, we used viral vectors to generate CCL2 overexpression in the adult brain. Seven weeks after intracranial injection of rAAV-CCL2, CCL2 was expressed at high levels in cortical and hippocampal neurons. Previous work has documented neuronal tropism for AAV vectors and long-term expression [35,68]. In response, two markers of microglial activation, CD45 and MHCII, were elevated around the site of injection. Both rounded and ramified morphologies were observed. CCL2 also increased expression of markers indicating both a proinflammatory M1 phenotype and an alternative M2 phenotype. Increased staining was observed for the M1 markers calgranulin B (S100A9) and IL-1β, as well as for the M2-selective markers arginase 1 and YM1. In addition, cytokine/chemokine production and gene expression data confirm that both M1 and M2 marker RNAs and proteins were induced. Together, these results indicate that CCL2 induces a unique profile of activation markers, thereby influencing the cerebral inflammatory environment. The dichotomy between M1 and M2 activation states appears blurry and may not be a useful categorization of microglial activation.

Microglia populate the CNS during early embryogenesis, whereas monocytes circulate in the blood, patrolling the environment. In untreated normal mice, monocytes are generally unable to enter the brain, which is anatomically separated from the periphery by the blood-brain barrier (BBB) [40,69]. The roles of peripheral monocyte subsets and their extravasation vary in different disease models and different organs [70]. Identification of monocyte infiltration has been greatly improved by the development of GFP/RFP knock-in reporter mice [71-73] and the use of GFP-labeled bone marrow chimeras following X-irradiation. However, potential effects of radiation on the brain microenvironment, including microglial activation, BBB integrity and promoting myeloid cell engraftment have been raised [74]. To track the rate of monocyte infiltration *in vivo*, we used the transfer of GFP+ bone marrow cells positively selected for the CD11b monocytic subset. We have previously shown that this monocytic subset migrates into APP/PS1 mouse brain in the absence of X-irradiation and become localized around amyloid plaques [40]. In the present study, we have shown that CCL2 was able to promote the extravasation of blood-derived GFP monocytes into wild-type mouse CNS. Immunohistochemistry revealed GFP+ cells in the parenchyma of the ipsilateral hemisphere in the vicinity of the injection site as well as in perivascular areas. These cells were round with a diameter approximating 10 μm, consistent with the size expected for monocytes. Stereological counts showed significantly more GFP+ cells in hippocampus than in anterior cortex. No GFP+ cells were observed in the contralateral hemisphere (cohort 1) in the absence of CCL2 overexpression. We also isolated single-cell suspensions from brain homogenates from cohort 2 for flow cytometry. GFP+ cells could be identified only in CCL2-treated brains. It is likely that neurons are not captured by this isolation procedure. Nineteen percent of the recovered cells were recruited GFP+ in the brains of CCL2-treated mice. Our data are in agreement with other findings showing that CCL2 is sufficient for recruiting blood-borne cells to sites of brain injury [75]. It is likely that the CCL2 levels achieved in this model may also be reached during periods of CNS inflammation. This model may be a useful experimental system to analyze the effects of CCL2 on extravasation of blood-borne macrophages across the BBB.We used immunofluorescence to determine whether recruited GFP+ cells express markers in common with microglia. We observed colocalization of GFP with YM1, but not with MHCII. Thus, it is not possible to discriminate resident microglia from infiltrating monocytes with the microglial markers in common use at present. The extent to which infiltrating cells can enter the brain in disease states and differentiate into macrophages and/or microglia is unclear. Future experiments could examine longer time periods to determine patterns of differentiation of the monocytes entering the CNS in response to CCL2.

Several transgenic CCL2 mouse models have been developed in an effort to understand the chemotactic and recruitment properties mediated by brain CCL2 *in vivo*. CCL2 expressed under the control of the myelin basic promoter caused prominent mononuclear cell infiltration in the brain [3,76]. These cells were characterized as F4/80, Mac-1 and CD45 leukocytes and were located largely in the perivascular space. Inflammatory stimuli (lipopolysaccharide (LPS) and pertussis toxin) promoted parenchymal infiltration of leukocytes. Similarly, the glial fibrillary acidic protein (GFAP) promoter was used to overexpress CCL2 in another line of mice. These mice developed age-dependent accumulation of leukocytes in the perivascular space and increased expression of CD45 [77]. Inflammatory signals or viral infection resulted in more severe disease and/or more rapid onset of symptoms [33,78,79]. Our data demonstrate that CCL2 expressed under the chicken β-actin promoter and introduced into the adult CNS via AAV9 resulted in parenchymal recruitment of GFP+ BMDCs, as well as local presence of ramified and amoeboid-activated cells expressing MHCII and CD45.

In our model, activated microglia displayed both M1 and M2 phenotypes in response to overexpression of CCL2, at least at the time of this investigation. On the basis of published work, we believe the CCL2 was overexpressed for the majority of the 7-wk treatment period. Other authors have demonstrated that a transgenic mouse that expressed CCL2 under the GFAP promoter developed pertussis-induced reversible inflammatory encephalopathy and that CCL2 directed a Th1-biased inflammatory reaction, as shown by high levels of TNF-α, INFγ and IL-2 in the CNS. In contrast, mice lacking CCL2 exhibit decreased severity of experimental autoimmune encephalomyelitis with diminished Th1 cytokine secretion in the CNS [10,33,77,80]. Other research highlights the importance of CCL2 in Th2-selective activation [32,81]. In a pulmonary granuloma model, overexpression of mouse CCL2 via recombinant adenovirus serotype 5 differentially altered the outcome of the immune responses toward the Th2 phenotype and was accompanied by increased production of IL-10 and IL-13 [82]. However, we found that the levels of proinflammatory cytokines in the hippocampi of CCL2 mice either were not significantly increased (TNF-α, IL-1β and IL-17) or were undetectable (IFNγ and IL-12p70). In addition, the levels of anti-inflammatory cytokines such as IL-4, IL-10 and IL-13 were slightly but not statistically significantly increased, suggesting a modest impact of CCL2 on the M2 activation state. Instead, CCL2 induced a pattern of cytokine induction that was unique and not completely consistent with either an M1 or an M2 pattern. We observed elevations in the levels of IL-6, GM-CSF and CCL11 (eotaxin 1) following CCL2 overexpression.

Using qRT-PCR, immunohistochemistry or multiplex assay, we have shown that CCL2 upregulated the levels of genes or gene products associated with both M1 and M2 microglial activation. For example, mRNA levels for arginase 1 (Arg1), chitinase 3-like-3 (YM1), eosinophil-associated ribonuclease A family (Ear-2) and chemokine 24 (CCL24) were significantly increased. Arg1 and YM1 protein were also elevated as assessed by immunohistochemistry. These observations are consistent with an M2 polarized activation response [59,64]. Other genes whose mRNA or protein expression was increased significantly included the M1-associated markers S100A8, S100A9, IL-1β and Sirpβ. S100A8 and S100A9 are putative ligands of the receptor for advanced glycation end products [49], a proinflammatory receptor that has been implicated in many chronic inflammatory diseases, including AD [83,84]. Other markers of M1 activation, such as TNF-α, iNOS and Marco, were not significantly elevated.

The phagocytic activity of microglia expressing S100A8 and Sirpβ proteins have been reported in acute and chronic inflammation [46,85]. It is still not clear whether CCL2 acts as a modulator of resident microglial phagocytosis or as a chemoattractant for peripheral macrophages to infiltrate the brain and accelerate clearance of pathological aggregates. Recent evidence derived using genetically modified animal models of amyloid deposition suggests that infiltrating macrophages reduce amyloid deposits [15-18,86,87]. Other studies have suggested a role of CCL2 in accelerating the development of oligomeric and soluble amyloid-β levels [19,88,89]. Our data demonstrate that CCL2 not only was able to activate resident microglia but also induced gene expression linked to both the M1 and M2 activation pathways. The presence of both classes of markers could demonstrate a continuum of microglial activation. For instance, increases in mRNA levels for markers of both M1 and M2 activation in animal models of AD have been reported, suggesting heterogenicity of these cells [27,90]. Induction of “classical” activation of microglia has been proven to be beneficial in the clearance of amyloid-β pathology [91]. On the other hand, a recent report by Heneka and collaborators demonstrated that NLRP3 inflammasome deficiency skewed microglial activation toward a M2 signature but also resulted in decreased amyloid-β deposits [92]. Meanwhile, activation of microglia via LPS- or CX3CR1-deficient microglia significantly induced tau pathology, possibly via IL-1/p38MAPK signaling pathway activation [93,94]. These findings underscore the need for further examination of the functional properties of M1 vs M2 microglial activation. Although we did not investigate how this inflammation correlates with the pathology related to a specific neurological disease, the role of CCL2-mediated inflammation in pathological settings needs to be clarified. Interestingly, increased CCL2 levels have been found in the brain, serum and cerebrospinal fluid of AD patients [20,95]. Further, in a recent study, Westin et al. reported that the CCL2 levels in the cerebrospinal fluid are correlated with faster cognitive decline in patients with early-stage AD [96]. Changes in the levels of CCL2 and its CCR2 receptor have been found in the brain tissue of patients with trauma, prion disease, cerebral malaria, HIV dementia, Parkinson’s disease and multiple sclerosis (see [67] for review).

In conclusion, our study confirms the role of CCL2 in promoting the activation of several molecules that may participate in altering the immune response. The finding that CCL2 can influence the direction of an immune response further indicates the complexity of inflammatory responses and the importance of this molecule. Our data suggest an important role of CCL2 in activating microglia and defining the CNS inflammatory milieu. It also highlights the use of gene therapy approaches to investigate the role of blood-borne myeloid precursors in CNS diseases.

## Abbreviations

AD: Alzheimer disease; BMDC: Bone marrow–derived cell; CCL2: Chemokine (C-C motif) ligand 2; GFP: Green fluorescent protein; MCP-1: Macrophage chemoattractant protein 1; rAAV-9: Recombinant adeno-associated virus serotype 9.

## Competing interest

The authors declare that they have no competing interests.

## Author’s contributions

MLS led the study and was involved in all aspects of it, from surgeries to data analysis, figure generation, data interpretation and manuscript preparation. JA participated in surgeries, bone marrow preparation, immunohistochemical staining and data acquisition. KN participated in CCL2 cloning and generated AAV9 viruses. DCL participated in performance and analysis of the RT-PCR technique. PR participated in tissue preparation, immunohistochemical/fluorescence staining and data acquisition. PM designed and supervised the stereological procedures. CC and XL assisted with multiplex chemokine/cytokine assay performance and analysis of data. DM and MNG were involved in the overall conception of the study, experimental design, data analysis and interpretation, and manuscript preparation, and they secured funding for the project. All authors read and approved the final manuscript.
